# Controlled Electromagnetically Induced Transparency and Fano Resonances in Hybrid BEC-Optomechanics

**DOI:** 10.1038/srep22651

**Published:** 2016-03-09

**Authors:** Kashif Ammar Yasir, Wu-Ming Liu

**Affiliations:** 1 Beijing National Laboratory for Condensed Matter Physics, Institute of Physics, Chinese Academy of Sciences, Beijing 100190, China

## Abstract

Cavity-optomechanics, a tool to manipulate mechanical effects of light to couple optical field with other physical objects, is the subject of increasing investigations, especially with regards to electromagnetically induced transparency (EIT). EIT, a result of Fano interference among different atomic transition levels, has acquired a significant importance in many areas of physics, such as atomic physics and quantum optics. However, controllability of such multi-dimensional systems has remained a crucial issue. In this report, we investigate the controllability of EIT and Fano resonances in hybrid optomechanical system composed of cigar-shaped Bose-Einstein condensate (BEC), trapped inside high-finesse Fabry-Pérot cavity with one vibrational mirror, driven by a single mode optical field and a transverse pump field. The transverse field is used to control the phenomenon of EIT. It is detected that the strength of transverse field is not only efficiently amplifying or attenuating out-going optical mode but also providing an opportunity to enhance the strength of Fano-interactions which leads to the amplification of EIT-window. To observe these phenomena in laboratory, we suggest a certain set of experimental parameters. The results provide a route for tunable manipulation of optical phenomena, like EIT, which could be a significant step in quantum engineering.

## Introduction

Cavity-optomechanics that couples coherent electromagnetic field with other mechanical objects via radiation pressure has become a significant area of research over the few years^1^,^2^. The demonstration of cavity-optomechanics with ultra-cold particles, like Bose-Einstein condensate (BEC), provides stunning manipulation of solid-state physics along with optical physics^3^,^4^. Through several investigations, different aspects of BEC have made spectacular contributions in understanding complex systems^5^,^6^,^7^,^8^,^9^,^10^,^11^,^12^. In optomechanical systems, coupling is obtained by radiation pressure^13^ and indirectly via quantum dot^14^ and ions^15^. Optomechanics helps mechanical effects of light to cool movable mirror to its quantum mechanical ground state^16^,^17^,^18^,^19^,^20^,^21^ and provides a platform to study strong coupling effects in hybrid systems^22^,^23^,^24^. Recent magnificent discussions and simulations on bistable behavior of BEC-optomechanical system^25^, high fidelity state transfer^26^,^27^, entanglement in cavity-optomechanics^28^,^29^,^30^,^31^,^32^, dynamical localization in field of cavity-optomechanics^33^,^34^ and coupled arrays of micro-cavities^35^,^36^ provide clear understanding for cavity-optomechanics.

Recently, electromagnetically induced transparency (EIT), a phenomenon of direct manifestation of quantum coherence^37^,^38^, has been extensively investigated and has provided a lot of remarkable applications^39^,^40^. In atomic system, EIT occurs due to Fano-interactions or quantum interference effects induced by coherently driving atomic wavepacket with an external pump laser field^41^,^42^. EIT effect has been theoretically investigated in optomechanical system^43^,^44^ and later experimentally verified in both optical^45^,^46^ and microwave^47^ domains. Fano resonances^48^, caused by Fano-interactions at different system configurations, have played an important role in understanding photo-electron spectra in atomic physics and have made a magnificent contribution in the latest field of plasmonics^49^. Fano resonances have also been investigated in hybrid cavity-optomechanics by using different configurations^50^,^51^.

In this report, we investigate the controlled behavior of electromagnetically induced transparency (EIT) and Fano Resonances in hybrid BEC-optomechanical system, by choosing similar method of control as proposed in ref. 52 where the tunable bistable dynamics have been discussed. The optomechanical system is composed of a cigar-shaped Bose-Einstein condensate (BEC) trapped inside high-finesse Fabry-Pérot cavity, with one fixed mirror and one moving-end mirror oscillating with frequency *ω*_*m*_ and maximum amplitude *q*_0_, driven by a single mode optical field *η* along the cavity axis and a transverse pump field *η*_⊥_. Transverse optical field is used to control the phenomenon of electromagnetically induced transparency (EIT) in the output probe laser field. By observing output probe field spectra, we show that the probe laser field can efficiently be amplified or attenuated depending on the strength of transverse optical field *η*_⊥_. Furthermore, we demonstrate emergence of Fano resonances by solving output field spectra, at different system detuning, and discuss controlled behavior of Fano resonances using transverse optical field. To observe these phenomena in laboratory, we have suggested a certain set of experimental parameters very near to the present experimental quests.

### BEC-Optomechanics

**Figure 1:**
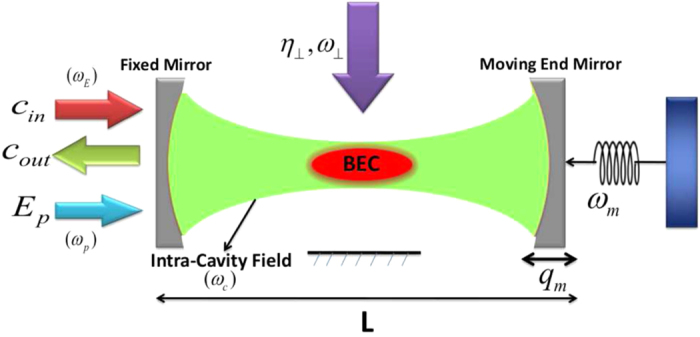
Cavity-Optomechanics with Bose-Einstein condensate. Cigar-shaped Bose-Einstein condensate (BEC) is trapped inside a Fabry-Pérot cavity of length *L*, with a fixed mirror and a vibrational-end mirror oscillating with frequency *ω*_*m*_ and maximum amplitude of *q*_0_, driven by a single mode external pump field with *ω*_*E*_. Intra-cavity field, with frequency *ω*_*c*_, interacts with atomic assemble and excites symmetric momentum side modes in condensed matter wave, due to photon recoil of intra-cavity field. The collective atomic excitation will mimic as an atomic mirror having similar characteristics to vibrational mirror of the system. To observe electromagnetically induced transparency (EIT) in output field spectra, we use external probe field with frequency *ω*_*p*_. *E*_*p*_ corresponds to the power of external probe field. The system is almost same as investigated by us in ref. 52 except the inclusion of external probe field which is crucial to investigate EIT. To produce control over the spectral behavior of out-going optical mode, we use another transverse field with intensity *η*_⊥_ and frequency *ω*_⊥_^63^.

We consider a Fabry-Pérot cavity of length *L* with a fixed mirror and a moving-end mirror driven by a single mode optical field with frequency *ω*_*E*_, as shown in Fig. 1. Inside the high-Q cavity, optical fields generate one dimensional optical lattice in which Cigar-Shaped BEC, containing N-two level atoms, is trapped^3^,^53^,^54^. Moreover, a transverse optical field, with strength *η*_⊥_ and frequency *ω*_⊥_, is used to to drive BEC perpendicularly, which scatters transverse photons to the intra-cavity field^52^,^55^,^56^,^57^,^58^. The vibrational end mirror performs harmonic oscillations under radiation pressure, with frequency *ω*_*m*_ and maximum amplitude *q*_0_, however, in the absence of intra-cavity radiation pressure, it performs Brownian motion due to external heat-bath.

The complete Hamiltonian of system can be divided into two parts, 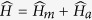, where 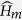 is related to the motion of side mirror inside the cavity and optical fields interacting with system, 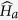 describes the atomic degree of freedom inside the system. The Hamiltonian 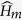 is given as^59^,


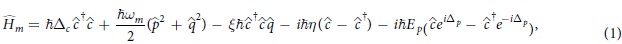


where 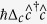 is energy of the intra-cavity field, with detuning Δ_*c*_ = *ω*_*c*_ − *ω*_*E*_ and frequency *ω*_*c*_. 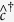 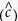 are creation (annihilation) operators for intra-cavity field interacting having commutation relation 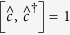. 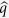 and 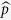 are dimensionless position and momentum operators, respectively, for vibrational end mirror having canonical relation 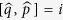, revealing scaled Planck’s constant *ħ* = 1. 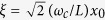 is the strength of coupling between viberating end mirror and intra-cavity optical field, where 
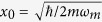
 is zero point motion mass *m* of the mirror. 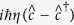 is the relation between intra-cavity field and external pump field 
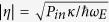
 with external pump field power *P* and intra-cavity field decay *κ*. Finally, last term describes external probe field and 
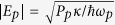
 is related to the power of the probe field. (Note: The system is nearly same as studied by us in ref. 52 except the addition of an external probe field which is crucial to investigate EIT). Δ_*p*_ = *ω*_*p*_ − *ω*_*E*_ is detuning between probe laser field frequency *ω*_*p*_ and external pump field *ω*_*E*_.

We consider the motion of BEC quantized along with the cavity-axis in strong detuning regime to adiabatically eliminate internal excited states of atoms and dilute enough so that atom-atom interaction effects can be ignored^56^,^57^. In addition, the Hamiltonian describing BEC and its association with cavity mode is written under momentum side-mode approximation (detailed derivation are shown in ref. 52) as,


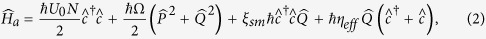


where 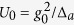 is the far off-resonant Rabi frequency with detuning Δ_*a*_ and *N* is the total number of bosonic particles in atomic mode. 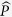 and 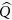 are dimensionless momentum and position quadratures, respectively, for atomic mode, with canonical relation 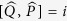, describing motion of atomic mode under mechanical effects of light with recoil frequency Ω = 4*ω*_*r*_ = 2*ħk*^2^/*m*_*a*_. 
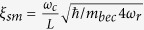
 is the coupling of atomic mode with intra-cavity optical field, where 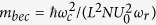 is side mode mass of condensate. Further, 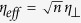 describes coupling of atomic degree of freedom with perpendicularly interacting optical field, where 
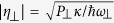
 is intensity of transverse field. From Eq. (2), it can be noted that in the absence of transverse optical field (i.e. when there is no excitation for *cos*(*kx*)) we recover same expression for atomic mode of cavity-optomechanics as in refs 3 and 25.

To incorporate the dissipation effects of intra-cavity field due to photon shot noises and thermo-mechanical noises effects associated with the motion of moving-end mirror and BEC via standard quantum noise operators, we derive Langevin equations from Hamiltonian *H*^60^.


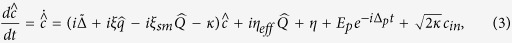



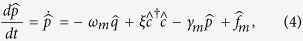



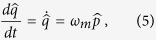



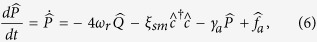



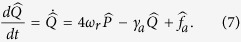


The term 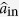 describes the Markovian input shot noise associated with intra-cavity field and 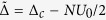 represents effective detuning of the system. *γ*_*m*_ is the decay rate of the moving-end mirror motion while 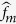 describes thermo-mechanical noise operator associated with Brownian motion of mechanical mode^61^,^62^. Further, *γ*_*a*_ and 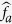 are damping of atomic mode due to harmonic trapping potential and 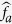 is Markovian noise operator connected with motion of atomic side modes, respectively. It should be noted that the Eqs (3, 4, 5, 6, 7) are same as derived in ref. 52 except the addition of external probe field in Eq. (3) to investigate EIT. We consider positions and momentum quadratures as classical variables to obtain steady state solutions of the Langevin equations. Further, we take optical field decay at its fastest rate so that time derivative can be set to zero in Eq. (3). The static solutions are given as,


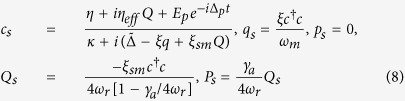


where *c*_*s*_, *q*_*s*_ and *Q*_*s*_ represent steady-state solution of intra-cavity field, the mechanical mirror displacement and the position of the BEC mode, respectively. As expected, the steady-state solutions given in Eq. (8) are same as derived in ref. 52 except the addition of external probe field relation in first equation.

To observe output field spectra, we deal with mean response of the system to probe field in presence of external pump field (control laser) and transverse field. First, we linearized quantum Langevin equations by inserting ansatz 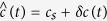, 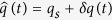, 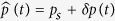, 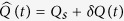 and 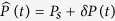 in Langevin equations and taking care of only first-order terms in fluctuating operators *δc*(*t*), *δq*(*t*), *δp*(*t*), *δQ*(*t*) and *δP*(*t*). The linearized quantum Langevin equations are now given as,


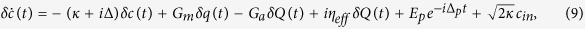



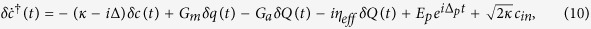



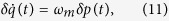



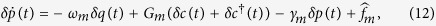



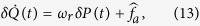



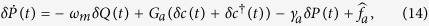


where 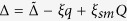 is the effective detuning of the system and 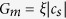, 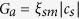 are the effective coupling of optical field with the moving-end mirror and the condensate mode, respectively. To solve mean value equation of the system, we write expectation value of operators in form 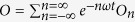, here *O* is a generic operator. 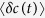, 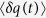, 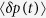, 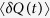 and 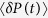 are the expectation values corresponding to fluctuating operators *δc*(*t*), *δq*(*t*), *δp*(*t*), *δQ*(*t*) and *δP*(*t*), respectively.

For the solution of linearized quantum Langevin equations, we assume that the coupling of external pump field *η* is much stronger than the coupling of external probe field *E*_*p*_. Under this assumption, the solutions of linearized Langevin equations can be approximated to the first order external probe field by ignoring all higher order terms of *E*_*p*_. Therefore, the solution for intra-cavity probe field is now given as,


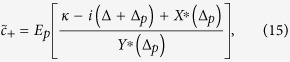



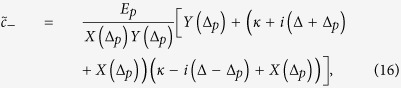


where


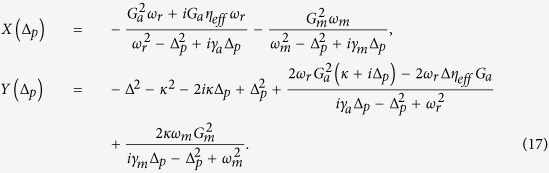


Eqs (15) and (16) clearly describe dependence of output field spectra on the coupling of different degrees of freedom. In particular, we can observe the rule of transverse optical field coupling with BEC mode in output field. Furthermore, to investigate EIT-like behavior, we write output field spectra by using input-output relation 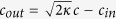
60, where *c*_*in*_ and *c*_*out*_ represent input and output field, respectively. Moreover, we ignore quantum noises associated with *c*_*out*_ and *c*_*in*_ as discussed earlier. The out-going optical field can be expressed as,


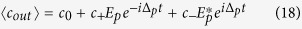


By using above relation and input-output field theory, we describe the components of output field spectra at probe field frequency as,


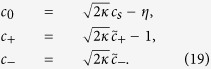


In order to examine EIT in output field, we define total out-going optical mode *E*_*T*_, at probe frequency *ω*_*p*_, as 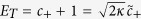, where *E*_*T*_ not only describes optical field leaking-out from cavity but also accommodates noise effects associated with the system. In absence of optical coupling with moving-end mirror and BEC mode, the output field spectra *E*_*T*_ at probe frequency will be reduced to,


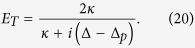


### Controllable EIT in Output Field

The hybrid BEC-optomechanical system shown in Fig. 1 is simultaneously driven by external pump field with frequency *ω*_*E*_ and probe field with frequency *ω*_*p*_, generating radiation pressure force which oscillates at frequency difference Δ_*p*_ = *ω*_*p*_ − *ω*_*E*_. When this resultant force resonates with frequency close to the frequency of mechanical mode *ω*_*m*_ or atomic mode (BEC) 4*ω*_*r*_, it gives rise to Stokes and anti-Stokes scatterings of light from the strong intra-cavity standing field. But Stokes scattering is strongly suppressed because, conventionally, optomechanical systems are operated in resolved-sideband regime 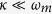, which is off-resonant with Stokes scattering and so only anti-Stokes scattering survives inside the cavity. Therefore, due to the presence of probe field and pump field inside system, Fano-interactions occur in mirror and atomic transition pathways which cause appearance of electromagnetically induced transparency (EIT) like behavior in output field spectra.

To make this study of tunable EIT and Fano resonances in hybrid BEC-Optomechanics experimentally feasible, we choose a regime of particular parameters very close to the recent experimental studies^1^,^3^,^54^. The EIT behavior in output field can only be observed when intra-cavity optical field is in stable regime. For this purpose, we follow similar stability conditions as developed and discussed in refs 52,55,57. We consider *N* = 2.3 × 10^4 87^*Rb* atoms trapped inside Fabry-Pérot cavity with length *L* = 1.25 × 10^−4^, driven by single mode external field with power *P*_*in*_ = 0.0164 *mW*, frequency *ω*_*E*_ = 3.8 × 2*π* × 10^14^ *Hz* and wavelength *λ*_*p*_ = 780 *nm*. The strength of external pump field is taken as *η*^2^/*κ*^2^ = 1.8. The intra-cavity optical mode oscillates with frequency *ω*_*c*_ = 15.3 × 2*π* × 10^14^ *Hz*, with decay rate *κ* = 1.3 × 2*π* *kHz*. Further, vacuum Rabi frequency of atomic mode is considered *U*_0_ = 3.1 × 2*π* *MHz* with detuning 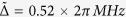. Intra-cavity field produces recoil of *ω*_*r*_ = 3.8 × 2*π* *kHz* in atomic mode trapped inside cavity with damping rate *γ*_*r*_ = 0.21 × 2*π* *kHz*. The moving-end mirror of cavity is considered as a perfect reflector oscillating with frequency *ω*_*m*_ = 1.02 × 2*π* *MHz* with damping *γ*_*m*_ = 1.1 × 2*π* *kHz*. From given parameters, one can observe that the system is in resolved-sideband regime because 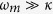, this condition is also referred to good-cavity limit.

**Figure 2:**
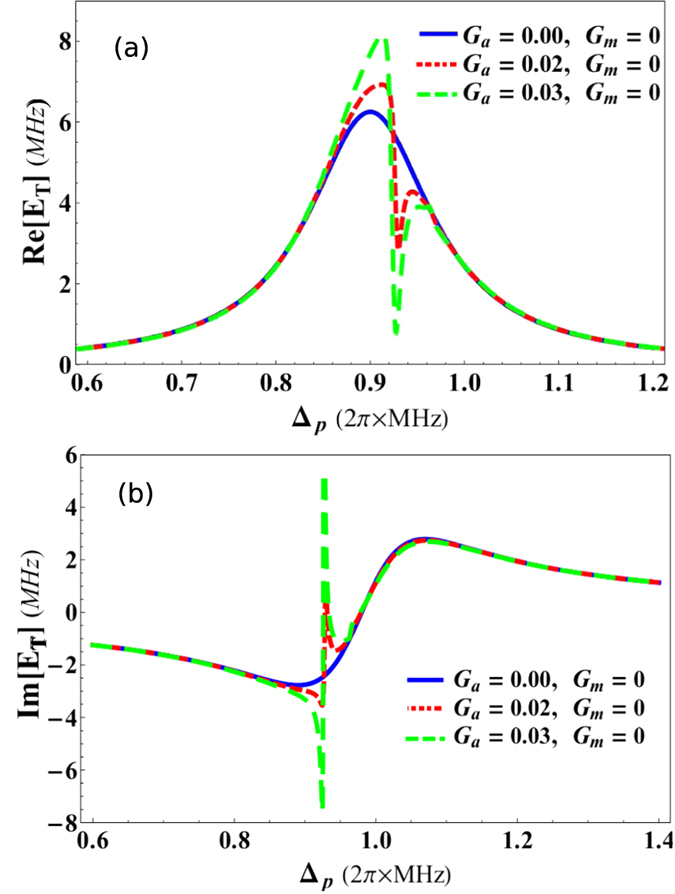
Single electromagnetically induced transparency in output field. The absorption (*Re*[*E*_*T*_]) and dispersion (*Im*[*E*_*T*_]) quadratures of the output field spectra *E*_*T*_ as a function of probe detuning Δ_*p*_/*ω*_*m*_ and in the absence of of transverse optical field coupling *η*_*eff*_/*κ* = 0. (**a**) Describes the behavior of single-EIT in real quadrature of output probe field with different atom-field and mirror field coupling strengths *G*_*a*_/*ω*_*m*_ = 0, *G*_*m*_/*ω*_*m*_ = 0 (blue curve), *G*_*a*_/*ω*_*m*_ = 0.02, *G*_*m*_/*ω*_*m*_ = 0 (red curve), and *G*_*a*_/*ω*_*m*_ = 0.03, *G*_*m*_/*ω*_*m*_ = 0 (green curve). (**b**) Contains similar behavior of imaginary quadrature of output probe field with different couplings *G*_*a*_/*ω*_*m*_ = 0, *G*_*m*_/*ω*_*m*_ = 0 (blue curve), *G*_*a*_/*ω*_*m*_ = 0.02, *G*_*m*_/*ω*_*m*_ = 0 (red curve), and *G*_*a*_/*ω*_*m*_ = 0.03, *G*_*m*_/*ω*_*m*_ = 0 (green curve). One can observe the emergence of single-EIT window with atom-field coupling due to the quantum interference among atomic transition pathways. The frequency of moving-end mirror is considered as, *ω*_*m*_ = 1.02 × 2*π* *MHz*. The atomic mode and mechanical mode damping rates are fixed to *γ*_*r*_ = 0.21 × 2*π* *kHz* and *γ*_*m*_ = 1.1 × 2*π* *kHz*, respectively. The cavity optical decay is taken as, *κ* = 1.3 × 2*π* *kHz*.

The real (*Re*[*E*_*T*_]) and imaginary (*Im*[*E*_*T*_]) quadratures of out-going probe field as a function of probe-cavity detuning are discussed in Fig. 2, in the absence of transverse field coupling *η*_*eff*_/*κ* = 0. Here, *Re*[*E*_*T*_] and *Im*[*E*_*T*_] accounts for in-phase and out-phase, respectively, quadratures of the output field spectra and also referred to absorption and dispersion behavior of out-going optical mode. Figure 2(a,b) demonstrate the single-EIT behavior of such absorption and dispersion quadratures, respectively, of output field spectra for different coupling strengths *G*_*a*_/*ω*_*m*_ = 0, *G*_*m*_/*ω*_*m*_ = 0 (blue curve), *G*_*a*_/*ω*_*m*_ = 0.02, *G*_*m*_/*ω*_*m*_ = 0 (red curve), and *G*_*a*_/*ω*_*m*_ = 0.03, *G*_*m*_/*ω*_*m*_ = 0 (green curve). To observe single-EIT behavior, we have considered case when system is only coupled with intra-cavity atomic mode or BEC and the coupling of moving-end mirror with intra-cavity optical mode is zero because the single-EIT behavior in cavity-optmechanics in presence of mirror coupling has already been discussed in previous works like^43^,^45^,^46^,^50^. We consider that the optomechanical system is being operated in strong coupling regime which means intra-cavity optical mode is strongly coupled to atomic mode of the system. When collective density excitation of atomic mode becomes resonant with intra-cavity optical mode, strong coupling between atomic and intra-cavity standing wave is generated. It is only possible when the strength of coupling between single atom and single photon of the cavity *g*_0_ = 10.9 × 2*π* *MHz* is larger than both decay rate of the atomic excited state *γ*_*r*_ = 0.21 × 2*π* *kHz* as well as intra-cavity field decay *κ* = 1.3 × 2*π* *kHz* 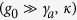
3,46. We can also note from mathematical expression of atomic coupling that the strength of atomic mode coupling is directly proportional to the vacuum Rabi frequency 
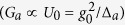
.

In Fig. 2, one can easily observe that there are no signatures of EIT in absorption and dispersion spectra of output field (blue curves) when intra-cavity field is not coupled with mechanical mode and condensate mode (*G*_*a*_/*ω*_*m*_ = 0, *G*_*m*_/*ω*_*m*_ = 0). While in red curve, the single-EIT window appears in output probe field due to intra-cavity optical mode coupling with atomic mode (BEC) (*G*_*a*_/*ω*_*m*_ = 0.02). However, coupling of intra-cavity field with mechanical mode is kept zero *G*_*m*_/*ω*_*m*_ = 0. Green curve shows the enhancement in single-EIT window in output probe field by increasing the coupling strength to *G*_*a*_/*ω*_*m*_ = 0.03. These results clearly prove the existence of single-EIT window in output probe field when intra-cavity optical mode is only coupled to atomic mode of cavity-optomechanics.

**Figure 3:**
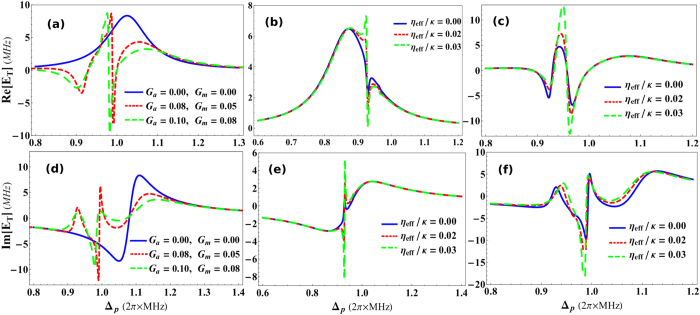
Controlled electromagnetically induced transparency with transverse field. The real (*Re*[*E*_*T*_]) and imaginary (*Im*[*E*_*T*_]) quadratures of the output probe field *E*_*T*_ as a function of probe detuning Δ_*p*_/*ω*_*m*_ and transverse optical field *η*_*eff*_/*κ*. (**a**,**d**) Accommodate double-EIT behavior in real and imaginary quadratures, respectively, in the absence of transverse field *η*_*eff*_/*κ* = 0 and with various coupling strengths *G*_*a*_/*ω*_*m*_ = 0, *G*_*m*_/*ω*_*m*_ = 0 (blue curve), *G*_*a*_/*ω*_*m*_ = 0.08, *G*_*m*_/*ω*_*m*_ = 0.05 (red curve), and *G*_*a*_/*ω*_*m*_ = 0.1, *G*_*m*_/*ω*_*m*_ = 0.08 (green curve). Similarly like atomic mirror, quantum interference in mechanical mirror transition paths cause the emergence of another transparency window in output field spectra. (**b**,**e**) Demonstrate single-EIT windows with coupling strengths *G*_*a*_/*ω*_*m*_ = 0.03, *G*_*m*_/*ω*_*m*_ = 0, as a function of transverse field strengths *η*_*eff*_/*κ* = 0 (blue curve), *η*_*eff*_/*κ* = 0.02 (red curve) and *η*_*eff*_/*κ* = 0.03 (green curve). Similarly, (**c**,**f**) show double-EIT behavior of output field spectra with coupling *G*_*a*_/*ω*_*m*_ = 0.1, *G*_*m*_/*ω*_*m*_ = 0.08, as a function of transverse field strengths *η*_*eff*_/*κ* = 0 (blue curve), *η*_*eff*_/*κ* = 0.02 (red curve) and *η*_*eff*_/*κ* = 0.03 (green curve). The transverse field scatters photons inside the cavity causing modification in both atomic as well as mechanical mirror transitions. The projection of these modifications on optical field leaking-out from cavity can be seen in given results. The remaining parameters used in numerical calculations are same as in Fig. 2.

Figure 3 describes the EIT behavior in the output field spectra of the optomechanical system in presence of probe detuning Δ_*p*_/*ω*_*m*_ and transverse optical field *η*_*eff*_/*κ*. Figure 3(a,d) represent absorption (real) and dispersion (imaginary) quadratures, respectively, of output probe field in the absence of transverse field *η*_*eff*_/*κ* = 0 with various coupling strengths *G*_*a*_/*ω*_*m*_ = 0, *G*_*m*_/*ω*_*m*_ = 0 (blue curve), *G*_*a*_/*ω*_*m*_ = 0.08, *G*_*m*_/*ω*_*m*_ = 0.05 (red curve), and *G*_*a*_/*ω*_*m*_ = 0.1, *G*_*m*_/*ω*_*m*_ = 0.08 (green curve). The results show that there are no signs of EIT-like behavior in absorption and dispersion spectra of output field (blue curves) when system is isolated form mechanical mode and condensate mode (*G*_*a*_/*ω*_*m*_ = 0, *G*_*m*_/*ω*_*m*_ = 0). On the other hand, in red curve, two EIT windows appear in output probe field because optical mode of the system is now coupled to both mechanical mode (moving-end mirror) (*G*_*m*_/*ω*_*m*_ = 0.05) as well as to condensate mode with coupling strength *G*_*a*_/*ω*_*m*_ = 0.08. Such behavior is also known as double-EIT response of output field^50^,^51^. When system is coupled to atomic mode and mechanical mode at the same time and optical mode of the system becomes resonant to both these modes, it gives rise to anti-Stokes scattering inside the system causing appearance of another EIT window in output field. Green curves in Fig. 3(a,d) demonstrate similar behavior double-EIT when the coupling strengths are increased to *G*_*a*_/*ω*_*m*_ = 0.1, *G*_*m*_/*ω*_*m*_ = 0.08. We observe that the quadratures of double-EIT behavior are increased by increasing coupling strengths. The given results in Fig. 3(a,d) show such double-EIT behavior in output field when optomechanical system is coupled to moving-end mirror of the system and BEC trapped inside the system.

Figure 3(b,e) demonstrate single-EIT behavior in absorption and dispersion quadratures, respectively, of output probe field under the influence of transverse optical field *η*_*eff*_/*κ* when intra-cavity optical mode is coupled to condensate mode with coupling strength *G*_*a*_/*ω*_*m*_ = 0.03 while the coupling of optical mode with moving-end mirror is zero *G*_*m*_/*ω*_*m*_ = 0. It should be noted that we cannot observe transverse optical field effects on single-EIT when intra-cavity optical degree of freedom is only coupled to the moving-end mirror, as shown in single-EIT results in previous works like^43^,^45^,^46^,^50^, because transverse optical field is only interacting with BEC trapped inside the cavity. Therefore, we only consider condensate mode coupling while studying effects of transverse field on single-EIT. Blue curves show single-EIT windows in output probe field in the absence of transverse optical field *η*_*eff*_/*κ* = 0. On the other hand, red and green curves demonstrate the effects of transverse field strengths *η*_*eff*_/*κ* = 0.02 and *η*_*eff*_/*κ* = 0.03, respectively, on the single-EIT behavior. When transverse field photon interacts with atomic mode of the system, it gives rise to the total photon number *n* inside the cavity by scattering transverse photons into the system, which give rise to the quantum interferences and directly enhance the EIT behavior in output field. We can observe such effects of transverse field in the results that the strength of single-EIT is efficiently amplified by increasing the strength of transverse optical field.

Similarly, Fig. 3(c,f) represent double-EIT behavior in absorption and dispersion quadratures respectively, of output probe field as a function of transverse optical field *η*_*eff*_/*κ* when intra-cavity optical mode is coupled to both condensate mode with coupling strength *G*_*a*_/*ω*_*m*_ = 0.1 and to the moving-end mirror is *G*_*m*_/*ω*_*m*_ = 0.08. Blue curves in both these figures describe double-EIT behavior in the absence of transverse field *η*_*eff*_/*κ* = 0. Besides, red and green curves represent double-EIT with transverse field strengths *η*_*eff*_/*κ* = 0.02 and *η*_*eff*_/*κ* = 0.03, respectively. We can observe, like single-EIT results, double-EIT windows are enhanced by increasing the transverse optical coupling. Therefore, in accordance to these results, we can confidently state that by increasing transverse optical field coupling, we can control the phenomenon of EIT in hybrid BEC-optomechanics.

### Tunable Fano resonances

The formation of Fano resonance in the output optical mode of hybrid optomechanical system is a fascinating phenomenon caused by quantum mechanical interaction between different degrees of freedom of the system^50^,^51^. The constructive and destructive quantum interferences among narrow discrete intra-cavity optical resonances are the foundations for Fano resonances in output field of such complex systems. The transverse field effects on EIT presented in Figs 2 and 3 are similar to the single and double-Fano resonances but tuned by transverse optical field. We conventionally observe Fano line shapes in EIT windows by tunning effective detuning of the system. The variation in effective detuning of the system brings modifications to the Fano-interactions, among atomic and mirror transition pathways, which causes shift in EIT window. Afterwards, we demonstrate Fano behavior of system output field with respect to different parameters.

**Figure 4:**
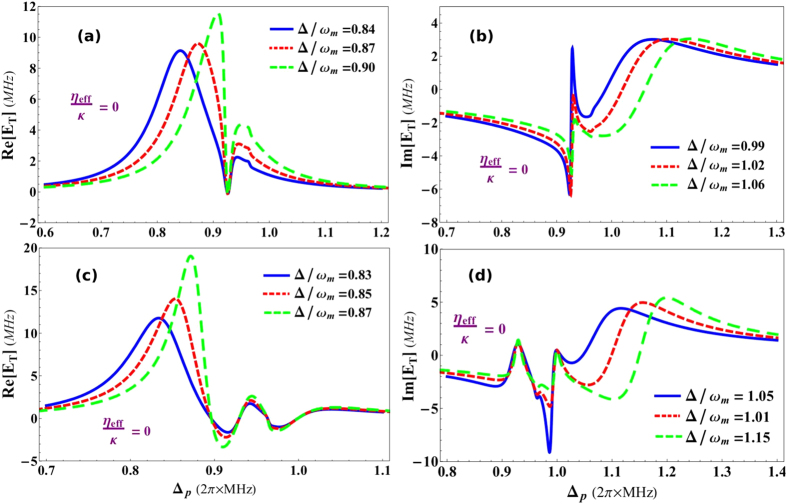
Fano resonance in output field. The behavior of Single-and double-Fano resonances in the absorption (real) and dispersion (imaginary) profile of output probe field in the absence of transverse field coupling, as a function of normalized probe field detuning Δ_*p*_/*ω*_*m*_ and normalized effective detuning of the system Δ/*ω*_*m*_. (**a**,**b**) Contain behavior of single-Fano resonance with coupling *G*_*a*_/*ω*_*m*_ = 0.03 and *G*_*m*_/*ω*_*m*_ = 0. (**a**) Demonstrates absorption spectra of output field with different values of effective detuning Δ/*ω*_*m*_ = 0.84 (blue curve), Δ/*ω*_*m*_ = 0.87 (red curve) and Δ/*ω*_*m*_ = 0.90 (green curve). Similarly, (**b**) shows dispersion behavior of output field with effective detuning Δ/*ω*_*m*_ = 0.99 (blue curve), Δ/*ω*_*m*_ = 1.02 (red curve) and Δ/*ω*_*m*_ = 1.06 (green curve). (**c**,**d**) Illustrate double-Fano resonances when the atom-field and mirror-field couplings are *G*_*a*_/*ω*_*m*_ = 0.1 and *G*_*m*_/*ω*_*m*_ = 0.08. (**c**) Shows real quadrature of output field under different effective detuning strengths Δ/*ω*_*m*_ = 0.83 (blue curve), Δ/*ω*_*m*_ = 0.85 (red curve) and Δ/*ω*_*m*_ = 0.97 (green curve). On the other hand, (**d**) presents imaginary quadrature of output field with effective detuning values Δ/*ω*_*m*_ = 1.05 (blue curve), Δ/*ω*_*m*_ = 1.1 (red curve) and Δ/*ω*_*m*_ = 1.15 (green curve). The Fano-interactions between atomic and mechanical mirror transition levels are generating resonances with different system detuning strengths, which can be seen in the results. Remaining parameters are same as in Fig. 2.

Figure 4 shows Fano resonances in the absorption (real) and dispersion (imaginary) profile of output probe field in the absence of transverse field coupling *η*_*eff*_/*κ* = 0, as a function of normalized probe field detuning Δ_*p*_/*ω*_*m*_ and normalized effective detuning of the system Δ/*ω*_*m*_. The coupling of intra-cavity optical mode with atomic mode is *G*_*a*_/*ω*_*m*_ = 0.03 while, the coupling of optical mode with mechanical mode is kept zero (*G*_*m*_/*ω*_*m*_ = 0) which means, optomechanical system is only coupled to the condensate mode trapped inside the cavity. Figure 4(a,b) describe absorption and dispersion profile, respectively, in output probe field as a function of normalized probe detuning. Blue curve in absorption shows Fano line with effective system detuning Δ/*ω*_*m*_ = 0.84. While, red and green curves in real quadrature represent fano behavior under the influence of effective detuning Δ/*ω*_*m*_ = 0.87 and 0.9, respectively. Similarly, blue curve in dispersion profile shows the existence of Fano line with effective system detuning Δ/*ω*_*m*_ = 0.99. Besides, red and green curves in imaginary quadrature of output field represent fano behavior under influence of effective detuning Δ/*ω*_*m*_ = 1.02 and 1.06, respectively. We can observe, each curve with different height follows a same dip in absorption and dispersion response which causes the formation of resonance in out-going optical mode.

**Figure 5:**
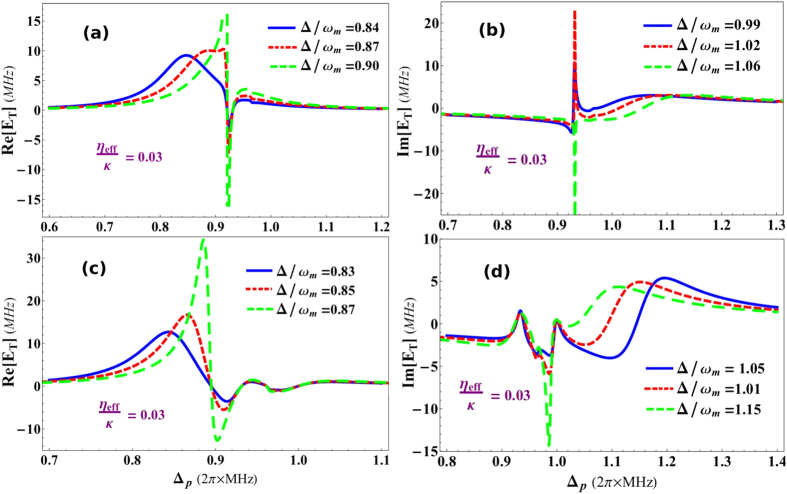
Transverse Field induced control over Fano resonances. The effects of transverse optical field, with coupling *η*_*eff*_/*κ* = 0.03, on Fano resonances in the absorption (real) and dispersion (imaginary) profiles of output probe field as a function of normalized probe field detuning Δ_*p*_/*ω*_*m*_ and under the influence of normalized effective detuning of the optomechanical system Δ/*ω*_*m*_. The transverse scatting of photons into the system will similarly alter the behavior Fano-interaction as it does to EIT. (**a**,**b**) Show transverse field effects on single-Fano resonance and the coupling strengths are same as in Fig. 4(a,b). (**a**) Shows absorption quadrature of output optical mode with different detuning strengths Δ/*ω*_*m*_ = 0.84 (blue curve), Δ/*ω*_*m*_ = 0.87 (red curve) and Δ/*ω*_*m*_ = 0.9 (green curve), while (**b**) accounts for imaginary quadrature of output field having effective detuning strengths Δ/*ω*_*m*_ = 0.99 (blue curve), Δ/*ω*_*m*_ = 1.02 (red curve) and Δ/*ω*_*m*_ = 1.06 (green curve). Similarly, (**c**,**d**) demonstrate the effects of transverse field on double-Fano resonance with same coupling strengths as in Fig. 4(c,d). (**c**) Shows absorption profile of output field under effective detuning strengths Δ/*ω*_*m*_ = 0.83 (blue curve), Δ/*ω*_*m*_ = 0.85 (red curve) and Δ/*ω*_*m*_ = 0.97 (green curve). On the other hand, (**d**) presents dispersion quadrature of output field with effective detuning values Δ/*ω*_*m*_ = 1.05 (blue curve), Δ/*ω*_*m*_ = 1.1 (red curve) and Δ/*ω*_*m*_ = 1.15 (green curve). All other parameters are same as in Fig. 2.

We further investigate the existence of double-Fano resonances in output probe field by introducing another coupling in the optomechanical system and modifing effective detuning^51^. As the phenomenon of EIT is very sensitive to the coupling with different degrees of freedom in the system, therefore, by introducing another coupling, we can convert single-Fano resonance to double-Fano resonance, as shown in Figs 4(c) and 5(d). The coupling of intra-cavity optical mode with moving-end mirror is *G*_*a*_/*ω*_*m*_ = 0.1 and the coupling of optical mode with condensate mode is *G*_*a*_/*ω*_*m*_ = 0.08. Figure 5(c) describes absorption and Fig. 5(d) describes dispersion profile in output probe field as a function of normalized probe detuning. Blue curves, in Fig. 5(c,d), show the double-Fano line with effective detuning values Δ/*ω*_*m*_ = 0.85 and 1.1, respectively. Similarly, red curves, in absorption and dispersion, accommodate the double-Fano response under the influence of effective detuning Δ/*ω*_*m*_ = 0.94 and 0.96, respectively and green curves account for the influence of effective detuning Δ/*ω*_*m*_ = 0.87 and 1.15, respectively. By analyzing these results, we come to know that the single-Fano resonance can be transformed to double-Fano resonances by coupling intra-cavity field with both atomic as well as mechanical degrees of freedom^50^,^51^.

In previous Fano resonance results, we have ignored the effects of transverse optical field coupling. However, it will be important to keep these effects and analyze the behavior of Fano resonances. Figure 5 illustrates such effects on Fano resonances emerging in output field spectra in the presence of transverse field coupling *η*_*e*_*ff*/*κ* = 0.03. Figure 5(a) shows real quadrature of out-going mode, containing single-Fano resonance, where blue, red and green curves correspond to the effective detuning strengths Δ/*ω*_*m*_ = 0.85, 0.87 and 0.9, respectively. On the other hand, Fig. 5(b) represents imaginary quadrature of output field, where blue, red and green curves correspond to the influence of system detuning strengths Δ/*ω*_*m*_ = 0.99, 1.02 and 1.06, respectively. One can observe, how quadratures of singe-Fano lines are increased due to the presence of transverse field. Transverse optical field causes scattering of photons inside the cavity which gives rise to intra-cavity photon number and this nonlinear factor brings modification to the out-going optical mode of cavity. It is understood that if we further increase the strength of transverse coupling, it will definitely further modify Fano behavior in output field.

Figure 5(c,d) show similar behavior for double-Fano resonances in real and imaginary profile of out-going probe field under the influence of transverse field strength. Figure 5(c) shows absorption and Fig. 5(d) shows dispersion behavior at transverse optical field strength *η*_*eff*_/*κ* = 0.03. Blue curves, in Fig. 5(c,d), show the effects of *η*_*eff*_/*κ* on double-Fano curves appearing in out-going mode with effective detuning values Δ/*ω*_*m*_ = 0.85 and 1.1, respectively. While, red curves, in absorption and dispersion quadratures, illustrate the effects on double-Fano response with effective detuning Δ/*ω*_*m*_ = 0.94 and 0.96, respectively and green curves account for the similar response double-Fano resonance under the influence of effective detuning Δ/*ω*_*m*_ = 0.87 and 1.15, respectively. By comparing results of Figs 4 with 5, we can easily note the effects of transverse optical field on the double-Fano resonance of the optomechanical system. The absorption and dispersion quadratures of single-Fano as well as double-Fano resonances are notably modified by increasing transverse optical field strength.

## Discussion

In conclusion, we discuss the controllability of electromagnetically induced transparency (EIT) and Fano Resonances in hybrid optomechanical system, by following same method as investigated in ref. 52. The system contains cigar-shaped Bose-Einstein condensate (BEC) trapped inside high-finesse optical cavity with one moving-end mirror and driven by a single mode optical field along the cavity axis and a transverse pump field. As the transverse optical field directly interacts with condensate mode which causes the scattering of transverse photon inside the cavity so, by varying transverse field, we can modify the dynamics of system. We have shown the controlled behavior of EIT in output probe field by using transverse field. We discuss existence of single-EIT window, in output field of cavity, in the absence of moving-end mirror which means intra-cavity optical mode was only coupled to atomic mode (BEC) of the system. The single-EIT as well as double-EIT windows, in out-going probe field, are efficiently amplified by increasing the strength of transverse optical field. Furthermore, single and double-Fano resonances are discussed in out-going probe field of the system. The transverse optical field shows similar effects on the emergence of Fano resonances as it does to the EIT. We have also suggested a certain set of experimental parameters to observe these phenomena in laboratory.

## Additional Information

**How to cite this article**: Yasir, K. A. and Liu, W.-M. Controlled Electromagnetically Induced Transparency and Fano Resonances in Hybrid BEC-Optomechanics. *Sci. Rep.* **6**, 22651; doi: 10.1038/srep22651 (2016).

## References

[b1] Kippenberg T. J. & Vahala K. J. Cavity optomechanics: Back-action at the mesoscale. Science 321, 1172 (2008). 18755966 10.1126/science.1156032

[b2] Aspelmeyer M., Kippenberg T. J. & Marquardt F. Cavity optomechanics. Rev. Mod. Phys. 86, 1391 (2014).

[b3] Brennecke F., Ritter S., Donner T. & Esslinger T. Cavity optomechanics with a Bose-Einstein condensate. Science 322, 235 (2008). 18787133 10.1126/science.1163218

[b4] Ritsch H., Domokos P., Brennecke F. & Esslinger T. Cold atoms in cavity-generated dynamical optical potential. Rev. Mod. Phys. 85, 553 (2013).

[b5] Choi D. & Niu Q. Bose-Einstein condensates in an optical lattice. Phys. Rev. Lett. 82, 2022 (1999).

[b6] Liu W. M., Wu B. & Niu Q. Nonlinear effects in interference of Bose-Einstein condensates. Phys Rev. Lett. 84, 2294 (2000). 11018868 10.1103/PhysRevLett.84.2294

[b7] Liu W. V. Theoretical study of the damping of collective excitation in a Bose-Einstein condensate. Phys. Rev. Lett. 79, 4056 (1997).

[b8] Lewenstein M. & Liu W. V. Optical lattices: Orbital dance. Nat. Phys. 7, 101 (2011).

[b9] Cai Z., Zhou X. & Wu C. Magnetic phases of bosons with synthetic spin-orbit coupling in optical lattices. Phys. Rev. A 85, 061605(R) (2012).

[b10] Cai Z., Wang Y. & Wu C. Frustrated Bose-Einstein condensates with noncollinear orbital ordering. Phys. Rev. B 86, 060517(R) (2012).

[b11] Dong L., Zhou L., Wu B., Ramachandhran B. & Pu H. Cavity-assisted dynamical spin-orbit coupling in cold atoms. Phys. Rev. A 89, 011602(R) (2014).

[b12] Hu H., Ramachandhran B., Pu H. & Liu X.-J. Spin-orbit coupled weakly interacting Bose-Einstein condensates in harmonic traps. Phys. Rev. Lett. 108, 010402 (2012). 22304247 10.1103/PhysRevLett.108.010402

[b13] Mancini S., Giovannetti V., Vitali D. & Tombesi P. Entangling macroscopic oscillators exploiting radiation pressure. Phys. Rev. Lett. 88, 120401 (2002). 11909431 10.1103/PhysRevLett.88.120401

[b14] Tian L. & Zoller P. Coupled ion-nanomechanical system. Phys. Rev. Lett. 93, 266403 (2004). 15697998 10.1103/PhysRevLett.93.266403

[b15] Naik A. *et al.* Cooling a nanomechanical resonator with quantum back-action. Nature 443, 193 (2006). 16971944 10.1038/nature05027

[b16] O′Connell A. D. *et al.* Quantum ground state and single-phonon control of a mechanical resonator. Nature 464, 697 (2010). 20237473 10.1038/nature08967

[b17] Teufel J. D. *et al.* Sideband cooling of micromechanical motion to the quantum ground state. Nature 475, 359 (2011). 21734657 10.1038/nature10261

[b18] Chan J. *et al.* Laser cooling of a nanomechanical oscillator into its quantum ground state. Nature 478, 89 (2011). 21979049 10.1038/nature10461

[b19] Liu K., Tan L., Lv C. H. & Liu W. M. Quantum phase transition in an array of coupled dissipative cavities. Phys. Rev. A 83, 063840 (2011).

[b20] Sun Q., Hu X. H., Ji A. C. & Liu W. M. Dynamics of a degenerate Fermi gas in a one-dimensional optical lattice coupled to a cavity. Phys. Rev. A 83, 043606 (2011).

[b21] Yi-Xiang Y., Ye J. & Liu W. M. Goldstone and Higgs modes of photons inside a cavity. Sci. Rep. 3, 3476 (2013). 24327105 10.1038/srep03476 PMC3858793

[b22] Groeblacher S., Hammerer K., Vanner M. R. & Aspelmeyer M. Observation of strong coupling between a micromechanical resonator and an optical cavity field. Nature 460, 724 (2009). 19661913 10.1038/nature08171

[b23] Teufel J. D. *et al.* Quantum mechanics: A light sounding drum. Nature 471, 204 (2011). 21390117 10.1038/471168a

[b24] Verhagen E., Deleglise S., Weis S., Schliesser A. & Kippenberg T. J. Quantum-coherent coupling of a mechanical oscillator to an optical cavity mode. Nature 482, 63 (2012). 22297970 10.1038/nature10787

[b25] Zhang K., Chen W., Bhattacharya M. & Meystre P. Hamiltonian chaos in a coupled BEC-optomechanical-cavity system. Phys. Rev. A 81, 013802 (2010).

[b26] Wang Y.-D. & Clerk A. A. Using Interference for High Fidelity Quantum State Transfer in Optomechanics. Phys. Rev. Lett. 108, 153603 (2012). 22587252 10.1103/PhysRevLett.108.153603

[b27] Singh S. *et al.* Quantum-state transfer between a Bose-Einstein condensate and an optomechanical mirror. Phys. Rev. A 86, 021801 (2012).

[b28] Abdi M., Pirandola S., Tombesi P. & Vitali D. Entanglement swapping with local certification: Application to remote micromechanical resonators. Phys. Rev. Lett. 109, 143601 (2012). 23083240 10.1103/PhysRevLett.109.143601

[b29] Abdi M., Pirandola S., Tombesi P. & Vitali D. Continuous-variable-entanglement swapping and its local certification: Entangling distant mechanical-modes. Phys. Rev. A 89, 022331 (2014).

[b30] Sete E. A., Eleuch H. & Ooi C. H. R. Light-to-matter entanglement transfer in optomechanics. J. Opt. Soc. Am. B 31, 2821 (2014).

[b31] Shi T., Jiang L. & Ye J. Phase sensitive two-mode squeezing and photon correlations from exciton superfluid in semiconductor electron-hole bilayer systems. Phys. Rev. B 81, 235402 (2010).

[b32] Dudarev A. M., Diener R. B., Wu B., Raizen M. G. & Niu Q. Entanglement generation and multiparticle interferometry with neutral atoms. Phys. Rev. Lett 91, 010402 (2003). 12906521 10.1103/PhysRevLett.91.010402

[b33] Yasir K. A., Ayub M. & Saif F. Exponential localization of moving-end mirror in optomechanical system. J. Mod. Opt. 61, 1318 (2014).

[b34] Ayub M., Yasir K. A. & Saif F. Dynamical localization of matter waves in optomechanics. Laser Phys. 24, 115503 (2014).

[b35] Ji A. C., Xie X. C. & Liu W. M. Quantum magnetic dynamics of polarized light in arrays of microcavities. Phys. Rev. Lett. 99, 183602 (2007). 17995407 10.1103/PhysRevLett.99.183602

[b36] Ji A. C., Sun Q., Xie X. C. & Liu W. M. Josephson effect for photons in two weakly linked microcavities. Phys. Rev. Lett. 102, 023602 (2009). 19257273 10.1103/PhysRevLett.102.023602

[b37] Scully M. O. & Zubairy M. S. Quantum Optics. Cambridge University Press (1997).

[b38] Safavi-Naeini A. H. *et al.* Electromagnetically induced transparency and slow light with optomechanics. Nature 472, 69 (2011). 21412237 10.1038/nature09933

[b39] Harris S. E., Field J. E. & Imamoglu A. Nonlinear optical processes using electromagnetically induced transparency. Phys. Rev. Lett. 64, 1107 (1990). 10041301 10.1103/PhysRevLett.64.1107

[b40] Boller K. J., Imamoglu A. & Harris S. E. Observation of electromagnetically induced transparency. Phys. Rev. Lett. 66, 2593 (1991). 10043562 10.1103/PhysRevLett.66.2593

[b41] Kash M. M. *et al.* Ultraslow group velocity and enhanced nonlinear optical effects in a coherently driven hot atomic gas. Phys. Rev. Lett. 82, 5229 (1999).

[b42] Lukin M. D. & Imamoglu A. Controlling photons using electromagnetically induced transparency. Nature 413, 273 (2001). 11565022 10.1038/35095000

[b43] Agarwal G. S. & Huang S. Electromagnetically induced transparency in mechanical effects of light. Phys. Rev. A 81, 041803(R) (2010).

[b44] Asjad M. Optomechanically dark state in hybrid BEC-optomechanical systems. J. Russ. Laser. Res. 34, 278 (2013).

[b45] Safavi-Naeini A. H. & Painter O. Proposal for an optomechanical traveling wave phonon-photon translator. New J. Phys. 13, 013017 (2010).

[b46] Weis S. *et al.* Optomechanically Induced Transparency. Science 330, 1520 (2010). 21071628 10.1126/science.1195596

[b47] Massel F. *et al.* Multimode circuit optomechanics near the quantum limit. Nat. Commun. 3, 987 (2012). 22871806 10.1038/ncomms1993 PMC3432470

[b48] Fano U. Effects of configuration interaction on intensities and phase shifts. Phys. Rev. 124, 1866 (1961).

[b49] Gallinet B. & Martin O. J. F. *Ab initio* theory of Fano resonances in plasmonic nanostructures and metamaterials. Phys. Rev. B 83, 235427 (2011).

[b50] Qu K. & Agarwal G. S. Fano resonances and their control in optomechanics. Phys. Rev. A 87, 063813 (2013).

[b51] Akram M. J., Ghafoor F. & Sair F. Electromagnetically induced transparency and tunable fano resonances in hybrid optomechanics. J. Phys. B 48, 065502 (2015).

[b52] Yasir K. A. & Liu W. M. Tunable bistability in hybrid Bose-Einstein condensate optomechanics. Sci. Rep. 5, 10612 (2015). 26035206 10.1038/srep10612 PMC4451843

[b53] Esteve J. *et al.* Squeezing and entanglement in a BoseEinstein condensate. Nature 455, 1216 (2008). 18830245 10.1038/nature07332

[b54] Brennecke F. *et al.* Cavity QED with a BoseEinstein condensate. Nature 450, 268 (2007). 17994093 10.1038/nature06120

[b55] Paternostro M., Chiara G. D. & Palma G. M. Cold-atom-induced control of an optomechanical device. Phys. Rev. Lett. 104, 243602 (2010). 20867301 10.1103/PhysRevLett.104.243602

[b56] Zhang J. M., Cui F. C., Zhou D. L. & Liu W. M. Nonlinear dynamics of a cigar-shaped Bose-Einstein condensate in an optical cavity. Phys. Rev. A 79, 033401 (2009).

[b57] Yang S. *et al.* Controllable optical switch using a Bose-Einstein condensate in an optical cavity. Phys. Rev. A 83, 053821 (2011).

[b58] Zhang X. F. *et al.* Rydberg Polaritons in a Cavity: A Superradiant Solid. Phys. Rev. Lett. 110, 090402 (2013). 23496692 10.1103/PhysRevLett.110.090402

[b59] Law C. K. Interaction between a moving mirror and radiation pressure: A Hamiltonian formulation. Phys. Rev. A 51, 2537 (1995). 9911870 10.1103/physreva.51.2537

[b60] Gardiner C. W. Quantum Noise (Berlin, Springer, 1991).

[b61] Paternostro M. *et al.* Reconstructing the dynamics of a movable mirror in a detuned optical cavity. New J. Phys. 8, 107 (2006).

[b62] Giovannetti V. & Vitali D. Phase-noise measurement in a cavity with a movable mirror undergoing quantum Brownian motion. Phys. Rev. A 63, 023812 (2001).

[b63] Dong Y., Ye J. & Pu H. Multistability in an optomechanical system with a two-component Bose-Einstein condensate. Phys. Rev. A 83, 031608(R) (2011).

